# Harm is all you need? Best interests and disputes about parental decision-making

**DOI:** 10.1136/medethics-2015-102893

**Published:** 2015-09-23

**Authors:** Giles Birchley

**Keywords:** Decision-making, Paediatrics, Newborns and Minors, Law, Minors/Parental Consent

## Abstract

A growing number of bioethics papers endorse the harm threshold when judging whether to override parental decisions. Among other claims, these papers argue that the harm threshold is easily understood by lay and professional audiences and correctly conforms to societal expectations of parents in regard to their children. English law contains a harm threshold which mediates the use of the best interests test in cases where a child may be removed from her parents. Using Diekema's seminal paper as an example, this paper explores the proposed workings of the harm threshold. I use examples from the practical use of the harm threshold in English law to argue that the harm threshold is an inadequate answer to the indeterminacy of the best interests test. I detail two criticisms: First, the harm standard has evaluative overtones and judges are loath to employ it where parental behaviour is misguided but they wish to treat parents sympathetically. Thus, by focusing only on ‘substandard’ parenting, harm is problematic where the parental attempts to benefit their child are misguided or wrong, such as in disputes about withdrawal of medical treatment. Second, when harm is used in genuine dilemmas, court judgments offer different answers to similar cases. This level of indeterminacy suggests that, in practice, the operation of the harm threshold would be indistinguishable from best interests. Since indeterminacy appears to be the greatest problem in elucidating what is best, bioethicists should concentrate on discovering the values that inform best interests.

## Introduction

Where medical decisions must be made for infants, English law directs decision-makers—primarily parents and doctors—to follow the best interests test. Yet judgments of best interests have long been criticised for their indeterminacy since they can be informed by a variety of values.^1^ Many bioethicists have also argued that parents, who must juggle their own needs, and potentially those of siblings as well, with those of the child, are poorly directed by the literal demand that they do what is best for the child.^2^ Of the many critiques that have arisen from such analysis is the claim that the best interests test should be mediated^3^ ^4^ (or perhaps replaced)^5^ with a harm threshold for overriding parental decisions. These critics maintain that a harm threshold more adequately reflects the wide range of discretion parents are customarily allowed in their behaviours towards their child, and guides professionals by indicating that only harmful parental actions justify intervention. But would a harm threshold really be more understandable and strike the correct balance between parental liberty and children's interests? English law already contains a harm threshold, perhaps overlooked by bioethicists because it is rarely used in determinations of medical best interests. The experiences of the English courts in applying the harm threshold suggest that it is considered significantly more evaluative than, and suffers from similar levels of indeterminacy to, the best interests test. This paper therefore argues that mediating best interests with a harm threshold is ill judged, and efforts should instead be spent on reducing indeterminacy by specifying the values that should guide best interests decisions.

## The harm consensus

Many commentators suggest that best interests is an opaque test^1^ ^4^ ^6^ ^7^ that, taken literally, places unfair demands upon parents who will face the paradox of simultaneously trying to maximise benefits to all of their children while somehow maintaining their own well-being.^2^ ^6–8^ In the face of these apparent problems with the best interests test, these commentators argue that a harm threshold should mediate (or perhaps replace) best interests.^3–7^ ^9^ Calls to replace the best interests test^5^ are ambiguous because it is not clear if it is meant to apply only to specific uses of the test. To keep things simple, I will concentrate on claims that the harm standard should mediate best interests, in other words a harm threshold should govern which cases we consider appropriate to be decided by the best interests test.

Although accepting that best interests is the best guide for decision-making on behalf of a child, Diekema argues that the harm threshold is superior to best interests when judging whether intervention is justified against parental refusals of medical treatment.^4^ ^9^ Elliston^6^ argues that intervention against parental decisions should only be made on the basis of harm, and only then if the choice that leads to those harms is unreasonable. Others suggest more specific uses for the harm threshold. Shah^7^ proposes a ‘secure child standard’ should govern parental decisions to enter children into research. Under this standard the courts should ‘defer to parental decision-making unless the child is exposed to some unjustified risk of significant harm’ (at 179). de Vos *et al*^3^ claim that parental requests for withdrawal of life-sustaining treatment should be allowed if they do not increase the risk of preventable harm. Gillam^10^ argues that harm indicates the scope of parental decision-making in situations of genuine dilemma. Indeed, a recent review^11^ suggests that there is widespread consensus among bioethicists that the harm threshold should determine the permissibility of overriding parental wishes. I dispute the usefulness of these conclusions. While avoiding harm has been convincingly claimed to be the single moral principle likely to be common to all cultures and philosophies,^12^ this is only likely because harm can be understood in numerous ways, and these will not always be consistent with one another. The use of the harm threshold in English law exemplifies this, and I shall discuss this in due course.

No critique of the harm threshold can ignore Diekema's^4^ seminal paper, which remains the most considered and widely referenced discussion of the issue. While I concentrate upon Diekema's analysis, my focus here is on developing a general critique of insufficiency of the harm threshold for overriding parental decisions.

## The harm threshold explained

Diekema considers that parenting children is a basic liberty right. He identifies the harm threshold first with Mill's^13^ assertion that the only circumstance in which personal liberty may be infringed by a community is to prevent harm to others, and second with Feinberg's^14^ argument that such infringement must be effective and the option of last resort. In order to further define the concept, Diekema links harm to a second concept, *basic needs*.^i^ While Diekema does not define these, basic needs are predicated upon Rawls’ natural primary goods, the things ‘a rational man wants whatever else he wants’ (at 79),^15^ and for children might form a notional list containing the minimum that will ‘enable children to embark … on the process of self-discovery, self-determination and self-fulfilment’ (at 42).^16^ Using Feinberg's contention that harm is the thwarting of an interest, Diekema argues that the existence of harms in children can be determined by the degree to which children's basic needs are provided for. Where a parent persistently fails to provide for a child's basic needs, harm is caused. Should these failures be significant and serious, intervention is warranted.

Diekema acknowledges that the non-self-evident nature of harm, even when coupled with terms like ‘significant’ and ‘serious’, means that the harm principle suffers from problems of indeterminacy.^ii^ Diekema counterargues that a threshold based upon harm better fits the point where intervention against parental wishes is justified in practice and is therefore less likely to cause confusion in clinicians than the obfuscatory language of best interests. Dresser,^5^ arguing along similar lines, makes this point succinctly when she argues that it is difficult to conceive that male circumcision for religious purposes serves the child's best interests, yet the purportedly harmless nature of male circumcision leads to its widespread acceptance in many cultures. While harm is nevertheless indeterminate, it is only fair to say that Diekema does not appear overly concerned with indeterminacy, for he states (at 253):

The biggest problem with a best interest standard is not its subjectivity, but that it represents the wrong standard. State intervention is not justified because a decision is contrary to the child's best interest, but because it places the child at significant risk of serious harm. Discussing the child's ‘best interest’ fails to focus on the relevant standard for determining when state action is justified. The harm standard focuses discussion in the proper place.^4^

Yet I suggest that Diekema, and others who have adopted his arguments, make a fundamental mistake. The challenge of indeterminacy is the central problem of the best interests test, and merely switching terminologies from best interests to harm does nothing to address this in any of the circumstances it has been proposed. Indeed, for reasons I explain below, the change in terminology is also unhelpful.

## The harm threshold criticised and defended

Diekema's contention that parental authority is a liberty right akin to any other exercise of autonomy is vulnerable to critique. The notion that personal liberty extends to *parental* autonomy—quite possibly a misuse of Mill's doctrine^iii^—conflicts with claims that children have rights on their own account. Such rights are often argued to extend beyond simple claims to physical integrity to at least leave some space for presumptive self-determination,^16^ ^17^ most cogently seen in Feinberg's^18^ argument that children have a right to an open future.^iv^ On such a basis Hester^19^ argues bioethicists should go further than simply preventing harm and specify some positive interests of children which we are obliged to protect. While he does not develop the argument, Diekema's specification of harm as damage to basic needs does not prevent us from making some positive specification of harms, which might deflect this critique somewhat.

A second argument advanced by a number of authors^4^ ^6^ ^7^ ^9^ is that the harm threshold is more readily understood than best interests by legislators, doctors and parents. This claim is problematic for two reasons. First, although harm may appear a readily understandable concept, the ready understandability hides the fact that judgements of harm, especially in complex ethical dilemmas, contain complex value judgments. For example, fatal withdrawal of treatment is a harm of indefinable proportions—perhaps catastrophic, or perhaps not even harm at all. While, for the sake of brevity, I accept without argument (here) that fatal withdrawal of treatment is sometimes justifiably in a patient's interests,^20^ such situations do not always sit well with the concept of harm as understood in the vernacular. This muddles the claim that harm is the correct threshold because it is simple enough to be generally understood and suggests harm is at least as problematic as best interests in this regard.^v^

This indicates a second, related, problem: harm may not capture all the considerations we need to capture to make a decision. This is problematic because if harm is the *only* concept on which attention can justifiably be focused, this invites any otherwise justifiable claims against interests which are not readily labelled as harms to nevertheless be labelled as harms. Such labelling inevitably transforms the harm threshold into a complex test when it is used in the wide variety of situations wherein the best interest test is currently employed. While I concede that, unembellished, claims of self-evident physical harm might provide one plausible standard to judge overriding a parental consent and refusal in some contexts (such as vaccination, intrasibling tissue donation and paediatric research)—albeit one that remains contestable on the basis of the non-self-evidence of parental rights—any appeal to clarity is lost once harm is the only gateway to the best interests test. Indeed, because English law contains a harm threshold, we have examples of these problems at first hand.^vi^

## The harm threshold in English law

English law uses a harm threshold in cases where a child may be removed from its parents.^vii^ Only if a child is being harmed or is at risk of harm will the court decide what home serves the child's best interests, removing the implication that the law will seek better-than-adequate parents for a child.^21^ Thus the law has similarities to the harm threshold envisaged by some commentators for medical decision-making,^viii^ and examining how the threshold operates in the courts highlights two potential problems. First, as I shall detail in a moment it seems arguable that judges see evaluative overtones in a conclusion that a parent is harming their child, which they do not (explicitly)^ix^ read into best interests. This indicates more general difficulties with a harm threshold when a decision to override parental consent appears necessary on grounds other than substandard care. This non-pejorative approach is generally taken because there is sympathy towards the parents’ motivation—for instance in disputes over the withdrawal or withholding of treatment or disputes motivated by the parents’ religious beliefs—and the label of substandard care appears insensitive to pluralism.^x^ Second, as we shall see shortly, when the harm threshold is used to address genuine dilemmas, judgments of harm become as indeterminate as judgments of best interests.

My first claim is that the courts view ‘harm’ as implicitly a more evaluative term than ‘best interests’. This is not to claim that the best interests test does not involve evaluation, but instead to argue that, as a legal term of art, a judgement that an action is against a child's best interests may appear less pejorative than a judgement that an action is harmful. The way the courts use harm to narrowly focus upon (defective) parental care, and reserve best interests to consider a much wider range of issues than the harm threshold gives reasons for thinking this is the case.

An indication of this is the general avoidance of the term ‘harm’ in English legal cases where the courts do not wish to indicate that parental care is substandard. For example, religious refusal to consent to life-saving blood products is not described as harm in contemporary cases.^xi^ While I acknowledge a variety of explanations for this approach—for instance application of the harm threshold is demanded by statute only in cases where the courts consider applications for care and supervision or residence and contact orders—an approach favouring the use of best interests under the inherent jurisdiction of the court has arguably arisen because of reservations about using the harm standard where the courts perceive a legitimate plurality of views.

If the aim is to respect parental privacy in parenting, it is of course sustainable to say that the pejorative connotations of the language used are moot since interference on any basis is problematic for the parents concerned. Yet the family courts regularly perceive that, as well as the parents concerned, they address standards of parenting in general,^xii^ and in this context concerns about language may gain more traction. It might nevertheless be argued that the lack of transparency in the best interests test make it a more problematic way to offer this more general guidance to parents. Yet this assumes that guidance on the basis of harms is more transparent, a claim I shall now dispute.

## The indeterminacy of harms

The indeterminate nature of harms raises a second problem that challenges claims that the harm threshold is more readily understandable than best interests. The definition of harm at section 31(9) of the Children Act 1989 as ‘ill-treatment or the impairment of health or development’ might appear clear and unambiguous. Yet, given that such a definition will include a wide range of circumstances, juridical use of the harm threshold has suffered from indeterminacy:^xiii^ for example, the question of whether a child's cultural background should affect an assessment of the seriousness of harm has been answered negatively and positively in different cases.^xiv^

A stark demonstration of the indeterminacy of harms is in a series of reports of baby trafficking cases *Haringey Local Authority v C,*^23^ *Re E*,^24^ *Re D*^25^ and *Re A*.^26^ These cases share a series of key features: all concern childless couples of African origin living in the UK who attend charismatic Christian churches. Conventional fertility treatments had failed these couples and all had sought unconventional treatments at clinics in Africa. Most admitted paying large sums of money for these treatments, after which many of the women had experienced phantom pregnancy (pseudocyesis). All had contacted their family doctors in the UK in the belief they were pregnant, and, if examined (one was not), were found not to be. All had undergone labour in African clinics, and were presented with babies who were removed by the state authorities when they returned to the UK. None of the women were DNA matches for their child, although all were nevertheless convinced the child was theirs. In every case their physical and emotional care of the child was thought to be exemplary. Despite these strong similarities, the courts reached differing conclusions about whether the harm threshold had been reached. Thus in *Haringey Local Authority v C*,^23^ while Ryder J believed that the ‘mother’ had been the victim of a cruel deception, he ruled the child was at risk of harms consisting of growing up with false beliefs that he was a miraculous birth, potentially experiencing devastating consequences if he discovered his true origin and of facing possible difficulties due to the lack of family medical history. The child was permanently removed from the ‘mother’ on this basis. While there is no public report on the outcome of *Re E*,^24^ the judge was unimpressed with the ‘mother’ as a witness, and her comments suggest a similar conclusion regarding harms was drawn.^xv^ Conversely, in *Re D*,^25^ the judge considered both parents to have been the victims of an elaborate scam. The case attracted significant attention and the media reported the couple had been allowed to keep the baby.^27^ ^28^ Most recently, a media report following *Re A*^26^ states the putative parents were allowed to keep the child,^29^ the judge in this case concluding that, although the child had been harmed, the harm was not inflicted by the couple.

Common law is open to interpretation, and I shall not argue that the judges in one case or another erred in their application of the harm threshold. Instead I claim that, wherever it is employed, the harm threshold can readily be used to reach opposing conclusions in very similar circumstances. This indicates the harm threshold suffers problems of indeterminacy of similar magnitude to the best interests test. For this reason, replacing or mediating the best interests test with a harm threshold is misguided; the real issue that bioethicists should focus upon is reducing indeterminacy.

## Reducing indeterminacy

Almost 30 years ago Moreno^30^ observed:

in the case of non-competent patients—those who never have been in a position to develop relevant preferences such as young children […] some best interests test, founded on the ethical principle of beneficence, seems required. But, in itself, the notion of best interests is vacuous, and so further principles must be invoked in its interpretation.

Rather than specify some of these principles, advocates of the harm threshold seek to simplify the best interests test. Diekema's^4^ tying of the threshold to the concept of basic needs is alert to this problem, since an ‘objective’ list of such welfare needs would answer this criticism—although reaching agreement on the specifics of such a list is problematic.^31^ However, harm does not describe all the parental behaviours that merit state intervention, and Diekema errs in suggesting that it does. ‘Harm’ is a catch-all term with which few could disagree. It captures the wrongness of hitting a child around the head with a saucepan or abusing a child for pleasure. Yet these situations do not represent genuine dilemmas when we would need the guidance of a harm threshold, since almost nobody would argue that such actions were permissible. It is in situations of genuine ethical complexity that a focus on harm becomes inadequate, and, if used in such situations, harm merely frames more complex ethical judgements. Once harm is invested with complexity, unless the complex rules it follows are explained, it becomes open to charges of indeterminacy. All we have done is rename the best interests test while dealing with none of its failings. Moreover, because harm is implicitly evaluative, it is problematic when there is genuine cause to intervene in parental behaviour, but no cause for public condemnation. In fact, I suggest that in these situations the technical language of best interests is actually helpful, precisely because it can house complex concepts without attaching condemnation.

The trigger and scope for intervention against parental wishes does of course vary depending upon the situation encountered. But it would be a mistake to view harm as the only consideration we should pay heed to in such cases. Issues such as refusal of vaccination, or the place of parental consent for children's entry into clinical trials raise issues of communal as well as child-centred responsibilities. Issues which balance one child's interests against another require a broad consideration of benefits to one party as well as harms to the other. Harm can be a valuable intuition pump that can lead us to consider important factors pertaining to a child's best interests. However, absent some list of other values, judging best interests and harms is inevitably subjective. The best response to this indeterminacy is not to attempt to *rename* the best interests test, but to identify the values *informing* best interests. It is likely that rather than a single monolithic value, there are many such values that apply more or less to different situations. Although identifying such values is therefore beyond the scope of this paper, examples abound. For instance, it is clear that many bioethicists, including many advocates of the harm threshold, consider that the child's interests can be subordinate to the interests of their parents.^4^ ^6^ ^7^ ^9^ Other theorists favour a broadly collectivist approach that requires a restriction of parental capacity to benefit as well as to disbenefit.^17^ ^32^ To publicise such positions in a decision about best interests allows transparency and offers grounds for challenge. To rehearse and debate the validity of such positions is genuinely informative of assessments of best interests in a way that adopting a harm threshold is not.

## Conclusion

Many commentators advocate a harm threshold to mediate the best interests test because they claim it is more determinate than the best interests test, and more clearly reflects the freedoms of parents to decide how and when to benefit their children. Diekema,^4^ the harm threshold's most convincing advocate, acknowledges that harm has similar problems with indeterminacy to best interests, but also argues that a harm threshold is better understood by clinicians than best interests, an argument that others have extended to the judiciary and to parents themselves.

While recognising the potential for a harm threshold to have uses as an intuition pump or a cross-cultural standard, this paper argues that where a harm threshold is used to determine intervention, it is inferior to the best interests test. I have used the way the harm threshold operates in English law to demonstrate it is insufficiently broad in its scope to be helpful in genuinely troubling cases without carrying with it significant elements of indeterminacy. Such complexity invalidates claims that the harm threshold is readily understandable in all cases. Furthermore, the language of harm is strongly evaluative, rendering it less suitable than the technicolegal language of best interests when intervening against well-intentioned parental behaviours without wishing to condemn such behaviours.

None of this mitigates the indeterminacy of the best interests test, and this paper has contended that this is the greatest challenge to deciding children's interests. Recourse to a harm threshold offers nothing to this project, and the focus of bioethicists should be upon identifying and mapping the values that (should) inform best interests decisions.

## Footnotes

i Lists of basic needs are rarely specified, and Rawls specified only an incomplete list which included health and vigour, intelligence and imagination. Basic needs (or their inverse, basic interests) may be criticised separately, although this is not the focus of this paper.

ii Thereby anticipating criticisms from those who feel that harm is too anaemic or plastic a concept.

iii Since Mill argued the state might place very great restrictions upon parenting and complains: “it is in the case of children, that misapplied notions of liberty are a real obstacle to the fulfilment by the State of its duties. One would think that a man's children were supposed to be literally, rather than metaphorically, a part of himself, so jealous is opinion of the smallest interference of law with his absolute and exclusive control over them; more jealous than of almost any interference with his own freedom of action: so much less do the generality of mankind value liberty than power” (at 88–89).^13^

iv Even those who dispute claims that children have rights tend to accept that children have interests.

v Albeit we have little evidence of how best interests are understood in this context.

vi Although my focus is on English law, my understanding is that other common law jurisdictions such as the USA and Australia use the harm threshold similarly in cases of child abuse or neglect.

vii The Children Act 1989 section 1 (3–4) specifies a harm threshold is to be used residence and contact orders, and care and supervision orders, respectively known also as section 8 orders and part 4 orders. The circumstances of which these orders may be sought is elaborated at section 31(2).

viii Indeed Elliston explicitly favours the extension of this model.^6^

ix A number of commentators argue that they do so implicitly, however I do not think this negates my argument, since I acknowledge the indeterminacy of best interests test is a problem.^22^

x Whether the care is indeed substandard depends upon one’s metaethical (and jurisprudential) stance.

xi Consider refusal of consent to blood in, for example, *Birmingham Children's NHS Trust v B & C (2014) EWHC 531 (Fam); M Children's Hospital NHS Foundation Trust v Mr & Mrs Y* (2014) EWHC 2651(Fam) N*HS v Child B, Mr and Mrs B* (2014) EWHC 3486 (Fam); Refusal of consent for HIV treatment represents a rare case that attracts the opposite approach e.g. *Re JA (A Minor)* (2014) EWHC 1135 (Fam). It could be convincingly argued that the approach here was due to the facts of the case.

xii Consider, for example, *Re H-B* (2015) EWCA Civ 389

xiii The persistent ambiguity of corporal punishment in English law is another fine example, although since it is usually outside the ambit of the harm threshold (being instead governed by the Children Act 2004, s58(3) and case law, most notably *R v H* (2001) 2 FLR 431) it would be digressive to include it in the main discussion.

xiv Compare *Re D (Care: Threshold Criteria)* (1998) Fam Law 656 and *A Local Authority v N* (2005) EWHC 2956 (Fam).

xv Parker J (at paragraph 3) states the harm threshold is ‘amply made out’ despite not forming part of the (fact finding) ruling.
